# Personality Traits and Sociodemographic Correlates in Saudi Arabia: A DSM-5 AMPD Criterion B Study Using the PID-5-BF

**DOI:** 10.3390/healthcare14020157

**Published:** 2026-01-08

**Authors:** Saleh A. Alghamdi, Renad Khalid Alqahtani, Nawaf Fahad Bin Othaim, Farah Fahad AL-Muqrin

**Affiliations:** 1 Psychiatry Department, College of Medicine, Imam Mohammad Ibn Saud Islamic University (IMSIU), P.O. Box 5701, Riyadh 11432, Saudi Arabia; kq.renad@gmail.com (R.K.A.); nweef92@gmail.com (N.F.B.O.); 2 King Fahad Medical City, P.O. Box 59046, Riyadh 11525, Saudi Arabia; farahalmuqrin66@gmail.com

**Keywords:** personality traits, PID-5 BF, sociodemographic factors, Saudi Arabia, cross-sectional study

## Abstract

**Introduction:** Personality disorders are enduring, maladaptive patterns that impair social and vocational functioning. The DSM-5 Alternative Model for Personality Disorders (AMPD) distinguishes Criterion A (personality functioning: identity, self-direction, empathy, intimacy) from Criterion B (maladaptive trait domains: negative affectivity, detachment, antagonism, disinhibition, psychoticism). We frame this study within Criterion B, supporting the use of a dimensional approach that complements (rather than replaces) normative models like the Five-Factor Model (FFM) and addresses cross-cultural gaps amid Saudi Arabia’s rapid sociocultural change such as the reforms associated with Vision 2030. Given Saudi Arabia’s collectivist orientation and evolving sociocultural norms under Vision 2030, the dimensional approach of the AMPD Criterion B offers a culturally sensitive lens for capturing personality pathology beyond Western-centric diagnostic models. **Aim:** We aimed to examine how PID-5-BF maladaptive trait domains vary across key sociodemographic factors in Saudi adults. **Subjects and Methods:** This was a quantitative, cross-sectional analytical study conducted among Saudi adults using the PID-5-BF Convenience sampling was performed via the dissemination of an online survey; we aimed for 377 participants and obtained 343 completed responses (~91% of the target sample). For trait assessment, we used the PID-5-BF; analyses compared domains across sociodemographic groups. **Results:** Females showed a higher negative affect; participants ≤ 30 years exhibited higher psychoticism than those >40; and single individuals reported lower detachment and psychoticism than their married peers. **Conclusions:** Gender, age, and marital status are associated with differences in maladaptive trait expression, supporting the need for culturally tailored screening and interventions in Saudi mental health services. These findings should be interpreted with caution given the fact that WhatsApp-based convenience sampling was used, which may bias the results as the respondents were more likely to live in urban areas, be educated, and be technologically proficient.

## 1. Introduction

Personality disorders, as defined in the DSM-5-TR, are enduring, inflexible patterns of inner experience and behavior that deviate markedly from cultural expectations; they can emerge in adolescence or early adulthood and lead to distress within or impairment of social, occupational, or other important areas of daily functioning [1]. These disorders are traditionally diagnosed using categorical models, which classify individuals into discrete diagnostic categories based on symptom thresholds. However, categorical approaches have been criticized for their limited diagnostic reliability, high comorbidity rates, and poor sensitivity to cultural variation.

To address these limitations, the DSM-5 Alternative Model for Personality Disorders (AMPD) was introduced, which adopts a dimensional framework for understanding personality pathology. The AMPD comprises two interrelated criteria: Criterion A, which evaluates impairments in personality functioning (including identity, self-direction, empathy, and intimacy), and Criterion B, which assesses maladaptive personality traits across five domains, namely negative affectivity, detachment, antagonism, disinhibition, and psychoticism [2,3]. While Criterion A captures the severity of dysfunction, Criterion B characterizes the style and expression of pathology through trait dimensions. Importantly, maladaptive traits are not synonymous with personality disorders; rather, they represent trait-level vulnerabilities that may or may not reach the threshold for clinical diagnosis [3,4]. Within this dimensional framework, establishing the applicability and structural coherence of maladaptive trait domains across diverse populations is essential for meaningful cross-group interpretation.

The dimensional approach of Criterion B is particularly relevant in non-Western contexts, where cultural norms may shape the expression of personality traits differently from Western diagnostic prototypes. Given Saudi Arabia’s collectivist orientation and evolving sociocultural norms under Vision 2030, the AMPD’s trait-based framework offers a culturally sensitive lens for assessing personality pathology [5,6]. Vision 2030, launched in 2016, has introduced sweeping reforms in education, employment, and gender roles, contributing to rapid sociocultural transformation. These changes may influence the manifestation of traits such as detachment, antagonism, and disinhibition, which are sensitive to interpersonal expectations and role transitions.

Despite the relevance of dimensional models, most personality research in Saudi Arabia has relied on the Five-Factor Model (FFM) to assess normative traits [7,8]. While the FFM provides a broad framework for general personality structure, it does not capture the maladaptive extremes emphasized in the AMPD. To our knowledge, no studies published in the literature have applied the PID-5-BF to Arabic-speaking populations in Saudi Arabia, although research using the full PID-5 has emerged in neighboring countries. This research gap limits our understanding of how maladaptive traits vary across sociodemographic groups in the region.

This study aims to address that gap by examining how PID-5-BF trait domains differ in terms of gender, age, and marital status in a sample of Saudi adults. Understanding these associations may inform culturally attuned screening, early intervention, and public mental health strategies. For example, elevated disinhibition is linked to health-risk behaviors, while increased psychoticism and detachment may signal vulnerability to psychosis [9,10]. Moreover, PID-5 trait levels have been shown to differ in terms of age and sex, underscoring the importance of demographic context in personality assessment [11].

Accordingly, the research question guiding this study is as follows: How do maladaptive personality trait domains, as defined by DSM-5 AMPD Criterion B and measured by the PID-5-BF, vary across gender, age, and marital status among Saudi adults?

Overall, this study contributes to the growing literature on dimensional personality assessment by applying the PID-5-BF within a Saudi context. It seeks to clarify the distribution of maladaptive traits across key sociodemographic variables and evaluate the psychometric properties of the Arabic PID-5-BF. These findings may support the development of culturally responsive mental health services and screening tools in the region.

## 2. Materials and Methods

### 2.1. Study Design and Setting

We conducted a cross-sectional analytical study in Saudi Arabia using the Personality Inventory for DSM-5—Brief Form (PID-5-BF) to assess DSM-5 AMPD Criterion B maladaptive trait domains. Data were collected from 1 to 31 August 2024, using an online questionnaire. The study procedures, instruments, and reporting align with best-practice recommendations for survey studies.

### 2.2. Participants, Eligibility, and Sampling

Eligibility criteria included adults aged 18 years or older of Saudi nationality, who were fluent in Arabic. The survey targeted adults from the general population and was not restricted to individuals with a diagnosed personality disorder. Participants were recruited using convenience sampling through online distribution channels, primarily via WhatsApp; therefore, the total number of individuals who received or viewed the invitation could not be determined. Individuals who were unable to provide informed consent or who faced language barriers were excluded.

A priori sample size estimation was made using a 95% confidence level and a 5% margin of error, yielding a target of 377 participants. Of the 377 invitations distributed, 343 individuals completed the survey, resulting in a response rate of approximately 91%. Although this represents a high participation rate, the final sample fell short of the calculated target, thereby reducing statistical power and increasing the risk of Type II error, particularly for detecting small effect sizes. This limitation is explicitly acknowledged and further addressed in Section 4, where its implications for precision and generalizability are considered.

### 2.3. Tool: PID-5-BF (AMPD Criterion B)

The Personality Inventory for DSM-5—Brief Form (PID-5-BF) is a 25-item self-report instrument developed by the American Psychiatric Association to operationalize Criterion B of the DSM-5 Alternative Model for Personality Disorders (AMPD). It assesses five maladaptive trait domains—negative affectivity, detachment, antagonism, disinhibition, and psychoticism—which reflect the core dimensions of personality pathology. Each domain comprises five items rated on a 4-point Likert scale, with higher scores indicating greater trait expression. The PID-5-BF has demonstrated robust internal consistency, factorial validity, and cross-cultural applicability in both clinical and non-clinical populations [12,13]. Its brevity and dimensional structure make it suitable for large-scale screening and research in diverse settings, including Arabic-speaking samples.

### 2.4. Arabic Translation and Cultural Adaptation

The PID-5-BF was adapted to Arabic using a standardized multi-step procedure consistent with international guidelines for test translation and cultural adaptation [14]. First, forward translation was conducted independently by two bilingual experts fluent in Arabic and English. Second, an expert panel comprising psychiatrists and clinical psychologists reconciled discrepancies and ensured conceptual fidelity. Third, back-translation was performed by a translator blinded to the original instrument to assess semantic equivalence. Fourth, the reconciled version underwent expert review to evaluate semantic, idiomatic, experiential, and conceptual alignment with the original English version.

To assess clarity and response burden, a pilot test was conducted with a small sample of Arabic-speaking adults (*n* = 25), representative of the target population. Feedback from the participants resulted in minor phrasing adjustments being made prior to field deployment. This rigorous adaptation process was designed to preserve the instrument’s psychometric integrity and ensure cultural relevance for use in Saudi Arabia.

### 2.5. Psychometric Evaluation (Arabic PID-5-BF)

We conducted a comprehensive psychometric evaluation of the Arabic PID-5-BF. Its internal consistency was assessed using Cronbach’s α, and McDonald’s ω was reported where appropriate to account for multidimensionality (Table 1). The test–retest reliability was evaluated using intraclass correlation coefficients (ICCs) over a two-week interval. Construct validity was examined via exploratory factor analysis (EFA), supported by Kaiser–Meyer–Olkin (KMO) sampling adequacy and Bartlett’s test of sphericity. The results of these reliability and item-level descriptive statistics (means, ranges, and averages) are summarized in Table 1.

To examine the hypothesized five-factor structure of the Arabic PID-5-BF, we conducted exploratory factor analysis (EFA) using principal axis factoring. Sampling adequacy was assessed via the Kaiser–Meyer–Olkin (KMO) test, and Bartlett’s test of sphericity was used to confirm factorability. Factors were extracted based on eigenvalues > 1 and examined for alignment with the intended PID-5-BF domains.

### 2.6. Procedures and Data Collection

The survey consisted of two structured sections: (1) sociodemographic variables, including age, sex, nationality, region of residence, marital status, employment status, and educational attainment; (2) the Arabic version of the PID-5-BF, assessing maladaptive personality traits. The instrument was administered electronically via a secure online platform. Participants were first presented with an electronic informed consent form outlining the study’s purpose, procedures, confidentiality safeguards, and voluntary nature. Only those who actively indicated that they agreed to partake were permitted to proceed.

Data collection was conducted over a one-month period, from 1 August 2024 to 31 August 2024. Following data acquisition, responses were screened for completeness and quality, and data cleaning procedures were applied. Statistical analyses were performed thereafter, and study finalization—including locking the analytic dataset and preparing the manuscript—occurred upon completion of all the preprocessing steps.

### 2.7. Statistical Analysis

The Statistical analyses were performed using IBM SPSS Statistics for Windows, Version 26.0 (IBM Corp., Armonk, NY, USA). The participants’ characteristics were summarized using descriptive statistics. Between-group comparisons of the PID-5-BF domain scores across sociodemographic strata (e.g., gender, age, marital status) were conducted using appropriate parametric or nonparametric tests, based on the distributional properties of each variable. Assumptions for normality were evaluated using the Kolmogorov–Smirnov test with Lilliefors correction, and homogeneity of variance was assessed prior to test selection. A two-sided significance threshold of α = 0.05 was prespecified. To account for multiple comparisons across trait domains and demographic subgroups, we applied false discovery rate (FDR) correction using the Benjamini–Hochberg procedure. Effect sizes with 95% confidence intervals were reported alongside *p*-values to convey both statistical significance and the magnitude of observed differences.

Although the planned sample size was 377, the final analytic sample comprised 343 participants. This shortfall modestly reduced statistical power, particularly for detecting small effect sizes. Nonsignificant findings are interpreted with caution, and the implications for precision and Type II error risk are addressed in Section 4.

### 2.8. Ethical Consideration

This study was conducted in accordance with the ethical principles outlined in the Declaration of Helsinki (2013 revision), and it received approval from the Institutional Review Board (IRB) of Imam Mohammad Ibn Saud Islamic University (IMSIU) (Project Number: 602L2024; approval date: 4 February 2024). The IRB reviewed and approved the study protocol, including the procedures used for obtaining informed consent, data confidentiality, and participant rights. Participation was entirely voluntary, and all respondents provided their informed consent electronically prior to completing the survey. No identifying information was collected, and all data were stored securely and analyzed in aggregate to ensure confidentiality.

## 3. Results

### 3.1. Psychometric Properties of the Arabic PID-5-BF

Internal consistency was high for the total scale (Cronbach’s α = 0.90) and acceptable across all five domains, including disinhibition (α = 0.77), negative affectivity (α = 0.77), detachment (α = 0.72), antagonism (α = 0.71), and psychoticism (α = 0.74). The values of McDonald’s ω were consistent with the α estimates, supporting reliability across trait domains. The test–retest reliability over a two-week interval yielded an intraclass correlation coefficient (ICC) of 0.85 for the total score (95% CI: 0.71–0.88), with domain-level ICCs ranging from 0.73 to 0.84, indicating satisfactory temporal stability. Exploratory factor analysis (EFA) supported the hypothesized five-factor structure. Sampling adequacy was confirmed (KMO = 0.88), and Bartlett’s test of sphericity was significant (*p* < 0.001), indicating suitability for the factor analysis. Five factors emerged with initial eigenvalues of 7.392, 1.999, 1.600, 1.345, and 1.167, collectively explaining 51.8% of the total variance (Table 2).

Factor loadings ranged from 0.502 to 0.770, exceeding the conventional 0.40 threshold for interpretability. Each item loaded most strongly on its intended domain, with no substantial cross-loadings. Communality values confirmed that each item’s contribution to its primary factor exceeded its loading on other factors by at least 20%, supporting factorial distinctiveness. These findings provide robust preliminary evidence for the construct validity of the Arabic PID-5-BF (Table 3).

Distributional properties of the PID-5-BF domain and total scores were examined as part of the psychometric evaluation. Kolmogorov–Smirnov tests with Lilliefors correction indicated statistically significant deviations from normality for all domain and total scores (*p* < 0.001). Given the large sample size and the use of averaged Likert-type domain scores, such deviations were expected and do not compromise meaningful interpretation of the scale’s psychometric performance. Detailed test statistics are presented in Table 4.

Confirmatory factor analysis (CFA) and measurement invariance testing were not conducted in this study.

### 3.2. Sociodemographic Characteristics of the Sample

Of the 377 invitations distributed, 343 participants completed the survey, yielding a response rate of 90.96%. The sample skewed younger, with 47.5% aged ≤ 30 years, 23.2% aged 31–40, and 29.3% aged > 40. Females comprised 56.3% of the sample (*n* = 193), and males 43.7% (*n* = 150). Educational attainment was high: 65.6% held a university degree, 22.7% reported school-level education, and 11.7% held postgraduate qualifications.

In terms of marital status, 54.2% were single, 42.6% were married, and 3.2% were divorced or widowed. Employment status was diverse: 38.5% were government employees, 35.9% were not currently working, 12.2% were students, 8.2% worked in the private sector, and 5.2% were retired. Geographically, the majority resided in the southern region (51.0%), followed by the middle region (38.2%), and then other regions (10.8%). Full demographic distributions of the participants’ sociodemographic characteristics are presented in Table 5.

### 3.3. PID-5-BF Descriptive Statistics

The total PID-5-BF score averaged 28.6 (range: 0–74), indicating substantial variability in maladaptive trait burden across the respondents. At the domain level, negative affectivity showed the highest mean score (7.1), followed by psychoticism (6.6), detachment (5.9), disinhibition (4.8), and antagonism (4.3). Item-level patterns were consistent with the domain-level summaries. Within negative affectivity, worry was notably elevated (e.g., “I worry about almost everything”, mean = 1.81), while within psychoticism, unusual thought content was frequently endorsed (e.g., “…others say are strange”, mean = 1.57). The disinhibition items reflected moderate endorsement (e.g., “I feel like I act totally on impulse”, mean = 1.03). The antagonism items showed higher endorsement for status-seeking (e.g., “People … less important than me”, mean = 1.37) than for callous or exploitative traits (means ≈ 0.56–0.66). Full item-level statistics, including minima, maxima, and dispersion, are presented in Table 6.

### 3.4. Associations Between Maladaptive Trait Domains and Sociodemographic Factors

Consistent with the AMPD Criterion B framework, several statistically significant between-group differences emerged. Females scored higher in terms of negative affectivity (mean = 7.5 vs. 6.7; *p* = 0.033), while males scored higher on antagonism (mean = 4.8 vs. 3.9; *p* = 0.004). No significant gender differences were observed for the total PID-5-BF score or the remaining domains. Age-related differences were observed for psychoticism, with younger participants (≤30 years) scoring higher than those >40 years (mean = 7.2 vs. 5.9; *p* = 0.008). Marital status was associated with multiple domains: married participants scored higher in terms of detachment (mean = 6.7 vs. 5.6; *p* = 0.012), psychoticism (mean = 7.5 vs. 6.0; *p* < 0.001), and the total PID-5-BF score (mean = 31.0 vs. 27.0; *p* = 0.009) compared with single individuals.

No statistically significant differences were observed for disinhibition across gender, age, or marital status. Furthermore, none of the PID-5-BF domains showed significant variation by education level, employment status, or region of residence (all *p* > 0.05). These findings are detailed in Table 7.

## 4. Discussion

The present findings suggest that maladaptive personality trait expression, as conceptualized within the DSM-5 AMPD Criterion B framework, varies meaningfully across key sociodemographic characteristics in a Saudi adult sample. Patterns observed across gender, age, and marital status indicate that demographic context may shape both the intensity and configuration of maladaptive traits. Rather than representing isolated differences, these patterns may reflect broader sociocultural influences, developmental processes, and role-related expectations that are particularly salient in rapidly changing social environments.

In this context, the study examined maladaptive personality trait domains using the PID-5-BF and identified several notable between-group differences. Females exhibited higher levels of Negative Affectivity, whereas males scored higher on Antagonism. Younger adults (≤30 years) showed elevated Psychoticism compared with older participants, and married individuals demonstrated higher Detachment, Psychoticism, and overall PID-5-BF scores relative to single participants. In contrast, no significant differences were observed for Disinhibition across sociodemographic strata, suggesting that this trait domain may be less sensitive to demographic variation in this population.

The present findings align with the existing literature on maladaptive personality trait expression, indicating that sociodemographic variation documented in both Western and non-Western samples is also evident within a Saudi adult population. In line with prior research, females exhibited higher negative affectivity, males showed elevated antagonism, younger adults demonstrated higher psychoticism, and marital status was associated with increased detachment and psychoticism. Taken together, these patterns reinforce the cross-cultural applicability of the AMPD Criterion B framework and underscore the utility of dimensional trait assessment for capturing context-specific expressions of personality pathology.

These findings are consistent with prior research indicating sex-linked differences in emotional and interpersonal traits. For example, borderline personality disorder (BPD)—a condition characterized by heightened negative affect and impulsivity—has been associated with sex-specific neuroendocrine patterns, including elevated cortisol awakening response in women and increased testosterone levels in men [15,16]. Notably, cortisol, but not testosterone, has been linked to anger and emotional dysregulation in women, highlighting potential biological pathways that may partially account for observed sex differences in maladaptive trait expression [16,17,18]. Taken together, these findings, along with evidence of sex-related differences in affect regulation and interpersonal sensitivity observed in dimensional personality models, highlight the significance of biological and sociocultural factors in influencing maladaptive trait expression.

Age-related variation in Psychoticism aligns with evidence that psychotic-like experiences fluctuate across the lifespan, often peaking during adolescence and early adulthood [19,20,21]. Similarly, marital status emerged as a meaningful correlate of maladaptive traits, particularly Detachment and Psychoticism, echoing prior findings suggesting that intimate relationships may both buffer against and exacerbate underlying personality vulnerabilities depending on relational dynamics and contextual stressors [22,23].

Collectively, these findings underscore the importance of culturally attuned screening and intervention strategies in Saudi Arabia. In light of the rapid sociocultural transformations associated with Vision 2030—including evolving gender roles, changing family structures, and increasing mental health awareness—dimensional personality assessment may offer a nuanced framework for identifying individuals at risk and tailoring public mental health services to population-specific needs.

Although Kolmogorov–Smirnov tests indicated statistically significant deviations from normality for all PID-5-BF domain and total scores, the magnitude of these deviations was small. Given the large sample size and the use of averaged Likert-type domain scores, such departures from normality are expected and unlikely to meaningfully affect the robustness of group-level comparisons. Accordingly, the observed findings are interpreted as reflecting substantive sociodemographic differences in maladaptive personality traits rather than artifacts of distributional irregularities.

Several limitations should be considered when interpreting these findings. First, the final sample size (*n* = 343) did not reach the a priori target (*n* = 377), potentially reducing statistical power and increasing the likelihood of Type II error, particularly for small effect sizes [24,25]. Second, reliance on online self-report measures may have introduced recall bias, social desirability effects, or inattentive responding, which could have influenced observed associations. Third, recruitment via WhatsApp-based convenience sampling may have disproportionately captured urban, educated, and technologically proficient individuals, thereby limiting generalizability to the broader Saudi population. Furthermore, because the survey was distributed via an open online link, the total number of individuals who received or viewed the invitation could not be determined, precluding calculation of response rates and raising the possibility of selection bias in observed sociodemographic differences. Fourth, the cross-sectional design precludes causal inference and may be subject to residual confounding from unmeasured sociodemographic or psychological factors.

Finally, although exploratory factor analysis supported the five-factor structure of the Arabic PID-5-BF, confirmatory factor analysis and measurement invariance testing were beyond the scope of the present study. The absence of these procedures limits conclusions regarding the robustness of the factor structure and its equivalence across demographic subgroups. Consequently, the psychometric evaluation of the Arabic PID-5-BF should be interpreted as preliminary, and future studies incorporating CFA and invariance testing are needed to strengthen confidence in its structural validity [14,26].

## 5. Conclusions

This study examined how maladaptive personality traits, as defined by DSM-5 AMPD Criterion B and measured via the Arabic PID-5-BF, vary across key sociodemographic factors in a sample of Saudi adults. Significant differences in terms of gender, age, and marital status were observed. For example, females scored higher in terms of negative affectivity, males on antagonism, younger adults on psychoticism, and married individuals on detachment and psychoticism. These findings highlight the relevance of trait-based dimensional models in capturing culturally nuanced patterns of personality pathology. While the Arabic PID-5-BF demonstrated acceptable reliability and factorial structure, further validation is warranted. Overall, the results of this study underscore the importance of integrating sociodemographic context into personality assessment and support the development of culturally responsive mental health screening tools in Saudi Arabia.

## 6. Implications and Recommendations for Future Research

To address the limitations identified in this study and strengthen future research on maladaptive personality traits in Arabic-speaking populations, several methodological and conceptual recommendations are proposed. Future studies should enhance representativeness by employing probability-based or stratified sampling strategies and diversifying recruitment channels through mixed-mode or multi-frame designs. Sample sizes should be increased based on updated power analyses to ensure adequate precision for detecting small-to-moderate effects. To mitigate reporting bias, future studies should implement validated attention checks and ensure anonymity, particularly for sensitive items. Pre-registration of primary outcomes, application of appropriate multiplicity control procedures (e.g., Benjamini–Hochberg false discovery rate), and consistent reporting of effect sizes with 95% confidence intervals will enhance transparency and interpretability. Multivariable models should be used to estimate independent associations, and longitudinal designs should be adopted where feasible to examine temporal dynamics and causal pathways. Additionally, confirmatory factor analysis (CFA) should be conducted to validate the five-factor structure and assess model fit using indices such as the Comparative Fit Index (CFI), Tucker–Lewis Index (TLI), and Root Mean Square Error of Approximation (RMSEA). Measurement invariance across gender, age, and clinical status should also be evaluated to ensure the scale’s applicability across diverse subgroups. Finally, the integration of Criterion A (Level of Personality Functioning Scale) is recommended to explore the interplay between personality functioning, maladaptive traits, and sociodemographic factors within the AMPD framework.

## Figures and Tables

**Table 1:** Internal consistency and test–retest reliability of the Arabic PID-5-BF.

Domain	Cronbach’s α	ICC (95% CI)	Mean	Range	Item Mean
Disinhibition	0.77	0.76 (0.71–0.77)	4.5	0–15	0.90
Negative Affect	0.77	0.74 (0.71–0.76)	7.1	0–15	1.42
Detachment	0.72	0.84 (0.77–0.87)	6.2	0–15	1.24
Antagonism	0.71	0.75 (0.73–0.79)	4.3	0–15	0.86
Psychoticism	0.74	0.73 (0.70–0.75)	6.6	0–15	1.32
Total Score	0.90	0.85 (0.71–0.88)	28.6	0–74	1.14

**Table 2:** Exploratory factor analysis: eigenvalues and variance explained.

Factor	Eigenvalue	Variance Explained (%)	Cumulative Variance (%)
1	7.392	29.57	29.57
2	1.999	7.99	37.56
3	1.600	6.40	43.96
4	1.345	5.38	49.34
5	1.167	2.46	51.80

**Table 3:** Rotated factor loadings of the Arabic version of the PID-5-BF.

Items	Factor 1	Factor 2	Factor 3	Factor 4	Factor 5
Disinhibition domain					
1. People would describe me as reckless.	0.717				
2. I feel like I act totally on impulse.	0.743				
3. Even though I know better, I can’t stop making rash decisions.	0.770				
5. Others see me as irresponsible.	0.502				
6. I’m not good at planning ahead.	0.543				
Negative Affect					
8. I worry about almost everything.		0.705			
9. I get emotional easily, often for very little reason.		0.740			
10. I fear being alone in life more than anything else.		0.543			
11. I get stuck on one way of doing things, even when it’s clear it won’t work.		0.677			
15. I get irritated easily by all sorts of things.		0.584		.	
Detachment					
4. I often feel like nothing I do really matters.			0.677		
13. I steer clear of romantic relationships.			0.772		
14. I’m not interested in making friends.			0.753		
16. I don’t like to get too close to people.			0.716		
18. I rarely get enthusiastic about anything.			0.591		
Antagonism					
17. It’s no big deal if I hurt other people’s feelings.				0.505	
19. I crave attention.				0.560	
20. I often have to deal with people who are less important than me.				0.577	
22. I use people to get what I want.				0.733	
25. It is easy for me to take advantage of others.				0.715	
Psychoticism					
7. My thoughts often don’t make sense to others.					0.503
12. I have seen things that weren’t really there.					0.504
21. I often have thoughts that make sense to me but that other people say are strange.			.		0.666
23. I often “zone out” and then suddenly come to and realize that a lot of time has passed.					0.726
24. Things around me often feel unreal, or more real than usual.					0.736

**Table 4:** Kolmogorov–Smirnov Normality Test Results: PID-5-BF Domain and Total Scores (*n* = 431).

Variable	K-S Statistic (D)	* df	*p*-Value
Negative Affectivity	0.069	431	<0.001
Detachment	0.073	431	<0.001
Antagonism	0.114	431	<0.001
Disinhibition	0.095	431	<0.001
Psychoticism	0.073	431	<0.001
PID-5-BF Total Score	0.061	431	<0.001

* df: number of valid cases (participants).

**Table 5:** Participants’ sociodemographic characteristics.

		*n*	%
Age (years)	≤30	162	47.5%
	31–40	79	23.2%
	>40	100	29.3%
Gender	Male	150	43.7%
	Female	193	56.3%
Education	School	78	22.7%
	University	225	65.6%
	Postgraduate	40	11.7%
Marital status	Married	146	42.6%
	Single	186	54.2%
	Divorced or widowed	11	3.2%
Employment	Student	42	12.2%
	Government	132	38.5%
	Private	28	8.2%
	Retired	18	5.2%
	Not working	123	35.9%
Residency	Middle	131	38.2%
	South	175	51.0%
	Others	37	10.8%

**Table 6:** Descriptive statistics of the PID-5-BF items.

Items	Min.	Max.	Mean	* SD
Disinhibition domain				
1. People would describe me as reckless.	0	3	0.91	0.98
2. I feel like I act totally on impulse.	0	3	1.03	0.97
3. Even though I know better, I can’t stop making rash decisions.	0	3	0.91	0.93
5. Others see me as irresponsible.	0	3	0.65	0.82
6. I’m not good at planning ahead.	0	3	1.02	0.91
Negative Affect				
8. I worry about almost everything.	0	3	1.81	0.99
9. I get emotional easily, often for very little reason.	0	3	1.67	1.00
10. I fear being alone in life more than anything else.	0	3	1.21	1.02
11. I get stuck on one way of doing things, even when it’s clear it won’t work.	0	3	1.22	0.92
15. I get irritated easily by all sorts of things.	0	3	1.19	0.92
Detachment				
4. I often feel like nothing I do really matters.	0	3	1.30	0.99
13. I steer clear of romantic relationships.	0	3	1.50	1.08
14. I’m not interested in making friends.	0	3	0.97	0.88
16. I don’t like to get too close to people.	0	3	1.22	0.96
18. I rarely get enthusiastic about anything.	0	3	1.11	0.92
Antagonism				
17. It’s no big deal if I hurt other peoples’ feelings.	0	3	0.56	0.74
19. I crave attention.	0	3	1.15	0.91
20. I often have to deal with people who are less important than me.	0	3	1.37	0.97
22. I use people to get what I want.	0	3	0.57	0.72
25. It is easy for me to take advantage of others.	0	3	0.66	0.80
Psychoticism				
7. My thoughts often don’t make sense to others.	0	3	1.56	0.91
12. I have seen things that weren’t really there.	0	3	0.96	0.97
21. I often have thoughts that make sense to me, but that other people say are strange.	0	3	1.57	0.96
23. I often “zone out” and then suddenly come to and realize that a lot of time has passed.	0	3	1.36	1.01
24. Things around me often feel unreal, or more real than usual.	0	3	1.15	0.95

* SD: Standard deviation.

**Table 7:** Association between PID-5-BF and sociodemographic characteristics of the participants.

	Negative Affect	Detachment	Antagonism	Disinhibition	Psychoticism	Total Score
Age (years)						
≤30	7.1	6.3	4.6	4.8	7.2	29.9
31–40	7.4	6.1	4.2	4.4	6.4	28.5
>40	6.9	5.8	4.0	4.2	5.9	26.8
*p*-value	0.617	0.424	0.287	0.320	0.008	0.144
Gender						
Male	6.7	6.4	4.8	4.8	6.8	29.4
Female	7.5	5.9	3.9	4.3	6.5	28.1
*p*-value	0.033	0.175	0.004	0.249	0.357	0.333
Education						
School	7.3	6.4	4.5	4.6	7.1	29.9
University	7.1	6.0	4.3	4.6	6.6	28.6
Postgraduate	6.6	5.8	4.1	4.0	5.6	26.1
*p*-value	0.602	0.551	0.713	0.542	0.091	0.293
Marital status						
Married	7.2	6.7	4.7	4.9	7.5	31.0
Single	7.0	5.6	4.1	4.3	6.0	27.0
Divorced or widowed	7.2	6.0	3.5	3.4	5.1	25.2
*p*-value	0.854	0.012	0.069	0.095	<0.001	0.009
Employment						
Student	6.8	6.1	4.8	4.6	6.6	28.9
Government	7.0	5.9	4.2	4.4	6.1	27.7
Private	7.1	6.0	4.2	4.3	7.1	28.7
Retired	7.7	6.3	5.2	5.3	7.6	32.0
Not working	7.2	6.3	4.2	4.5	6.9	29.1
*p*-value	0.897	0.948	0.530	0.870	0.154	0.695
Residency						
Middle	6.8	6.2	4.1	4.5	6.4	28.0
South	7.2	6.0	4.5	4.5	6.7	28.9
Others	8.0	5.8	4.1	4.7	6.9	29.6
*p*-value	0.167	0.758	0.428	0.929	0.658	0.740

## Data Availability

The data presented in this study are available on request from the corresponding author. The data are not publicly available due to institutional privacy policies and ethical restrictions related to participant confidentiality.
